# A cross-sectional study on the practice of wet nursing among *Muslim* mothers

**DOI:** 10.1186/s12884-021-03551-9

**Published:** 2021-01-21

**Authors:** C. A. R. Norsyamlina, H. Salasiah Hanin, A. M. Latifah, K. Zuliza, M. H. Nurhidayah, S. Rafeah, B. Nora’inan, I. Muhamad Zariff, A. Noor Ani

**Affiliations:** 1 Institute for Public Health, National Institutes of Health, Ministry of Health Malaysia, Shah Alam, Selangor Malaysia; 2 Faculty of Islamic Studies, Universiti Kebangsaan Malaysia, Bangi, Selangor Malaysia; 3 Academy of Contemporary Islamic Studies, Universiti Teknologi MARA, Shah Alam, Selangor Malaysia; 4 Faculty of Syariah and Law, Kolej Universiti Islam Antarabangsa Selangor, Kajang, Selangor Malaysia

**Keywords:** Wet nursing practice, Breastfeeding, Documentation, Islamic jurisprudence

## Abstract

**Background:**

Breastfeeding and wet nursing have been synonymous since ancient times. The practice of wet nursing of another woman’s child in Malaysia is on the rise due to the emergence of awareness among the public about the importance and advantages of breast milk. However, problems arise when there is no systematic system to record and trace the milk mother and milk child data, especially for Muslim participants as milk kinship could affect their relationship status in Islam. Therefore, this study aims to determine the practice of wet nursing among Muslim mothers in Selangor. Simultaneously, this study intends to provide the authorities with an accurate picture of the more aggressive compilation of steps to prevent duplication of consanguinity in wet nursing.

**Methods:**

This cross-sectional study was conducted on 100 women who had breastfed another child in Selangor. Data were obtained using a validated questionnaire (Cronbach alpha = 0.8) and processed using the SPSS software.

**Results:**

Results showed 43.0% of respondents had at least breastfed one someone else’s child. Meanwhile, there were 3.0% of the respondents were nursing seven to ten other children. A total of 237 children have been breastfed by the respondents (*n* = 100). Of these, 21.5% children were breastfed less than five times, while 78.5% children were breastfed less than five times. Most mothers recorded their milk child background data, and this shows that the community is aware of the importance of data documentation and it indirectly proves that the authorities should act on these current needs.

**Conclusions:**

This study shows that there is a wet nursing practice in the society. Obviously, a phenomenon, trend and practice in the society has the ground and basis as to why it existed and is upheld. Researches related to wet nursing and matters connected to it should continue so as to bring about much good to society.

**Supplementary Information:**

The online version contains supplementary material available at 10.1186/s12884-021-03551-9.

## Background

The practice of breastfeeding is influenced by both medical and socio-cultural factors. It has many aspects of anthropology’s “power structure” that find their expression in breast milk and practices that formed around it, socially, scientifically, and legally. It is widely accepted that breastfeeding has been recognized as the best method to provide nutrition for babies during their infancy stage of life (birth to 2 years old). Breast milk contains all the vitamins, minerals, and other nutritional elements that are appropriate to the needs of the baby [1–5]. Since early 1970s to the present time, the importance of breastfeeding exclusively by a biological has attracted much attention from medical practitioners, academic researchers and local community [6]. In cases in which the mother is unavailable or her milk supply is insufficient, the World Health Organization recommends that the milk from of another woman is preferable to artificial baby milks, particularly in emergency situations [7]. In short, if breastfeeding by a biological mother is not an option, the next preferred option is relactation (re-establishment of the milk supply) or wet nursing by another woman, followed by the least preferred option of using artificial infant milk.

The term “wet nursing” is used to portray whatever form of breastfeeding provided by someone other than the infant’s biological mother [8]. This practice does not raise any personal issues, but it involves the development of civil society because such practices can be convicted of consanguinity between children breastfed by a wet nurse and her family, as specified by the legislation. This includes issues related to sharia law and responsibilities, milk kinship that interferes with matters involving marriage, genitalia, wages of breastfeeding mothers, and various other legal aspects of breastfeeding [9]. Throughout the history of mankind, the provision of wet nursing service has always been a point for conversation or discussion whenever the subject of infant feeding practice comes up. However, the concept of wet nursing still exists today and is known as co-feeding or milk-sharing. The term clearly defines an activity of sharing mother’s milk or the sharing of expressed breast milk [1]. Its focus has changed towards the intention of donating as well as offering help within the community.

In Malaysia, the practice of breastfeeding has become more common due to awareness on the importance of breastfeeding. This practice is also in line with the development of the practice of wet nursing [10], where a certain number of mothers in Malaysia share their breast milk with children of different mothers for their nourishment [11]. Breastfeeding by a wet nurse also gives rise to several rules in Islam known as *Hukum Tahrim*, which means that wet nursing creates impediments to marriage between a nurse and her nursling, as well as between male and female (strange) nurslings suckling from the same nurse [8]. This means when a baby is being breastfed by a woman other than his or her biological mother for five times or more within the period of the first two-years, the baby then becomes her milk son or milk daughter. Therefore, the mother becomes *mahram* or *haram* (illegal) to be wedded to the child she so nursed [12]. In a study conducted by researchers from Turkey, 55.4% of the mothers considered wet nursing as beneficial. The main reason that affects their opinions is religious beliefs, in which they thought that wet nursing is a good deed. In addition, wet nursing is seen to be beneficial, not only for children but also mothers [13].

Milk kinship is created through breastfeeding a non-biological infant that results in a relationship similar to blood relationship. In Islam, children who consume milk from the same woman are forbidden or *haram* to wed each other. If, by chance, the children get married and it later becomes known that they were breastfed by the same woman, the marriage is annulled [14, 15]. A summary of the implications of milk kinship in this context is provided elsewhere [8, 14, 16, 17]. Milk kinship was also observed in the other cultures in Eastern Mediterranean. For example, in the early twentieth century in Greece, children receiving milk from the same mother were precluded from marrying each other [18]. The term milk sibling, or milk sister/brother, is also used by Anglo-Celtic Australian mothers in a non-religious context to describe the bond between their children when breastfeeding is practiced on a regular basis, often with family members or close friends, or when a woman breastfeeds a foster baby as well as her own child [8, 19].

Breast milk sharing is not an issue in the society in Malaysia and around the world. In fact, the establishment of human milk bank has been accepted and practiced in the West. Besides, all the writings and researches done in Malaysia are focused on the technical implementation of breastfeeding in in accordance with Islam. There is no specific evidence from the study that describes the practice of wet nursing, according to Islam. Therefore, researchers aim to determine the practice and processes implemented by Muslim mothers in practising wet nursing in the context of the society in Selangor. The findings of this study are expected to provide an accurate picture to the authority in drawing up measures more aggressively to avoid duplication of consanguinity as breastfeeding, and it is reasonable to enforce the practice of wet nursing recorded and monitored in a systematic and formal. It is also hoped that this research will eventually prompt the studies of wet nursing practice in the other states in Malaysia as well.

## Methods

### Study design and setting

This was a cross-sectional descriptive study that was carried out from January 2019 to July 2019. The study was conducted in nine districts (Sabak Bernam, Hulu Selangor, Kuala Selangor, Kuala Langat, Sepang, Hulu Langat, Gombak, Petaling and Klang) in Selangor (purposeful sampling of one of the states in Malaysia). The study was carried out a population of muslim women aged 21 to 50 years old using a purposive sampling method. A purposive sample is a non-probability sample that is selected based on the characteristics of a population that may fulfil the objectives of the study. Individuals who have consented to participate and had rich information were the targets hoping that they could provide more extensive data.

### Sample and recruitment

Data was collected using self-administered questionnaire via Google form. Researchers developed a questionnaire survey to determine the practice and processes implemented by Muslim mothers in practising wet nursing to other children in the context of the society in Selangor (please refer supplementary file). The questionnaire consisted of two parts: sociodemographic characteristics, and women’s practice on wet nursing. The inclusion criteria of respondents were i) Mothers who breastfeed other children (atlest one child); ii) Mother resident in Selangor; iii) Mother aged below 50 years old; iv) Consented to participate. The exclusion criteria were: i) Non-Muslim respondents; ii) Muslims outside of Selangor or living in other Malaysian states.

### Pilot test

A pilot study on 29 wet nursing muslim mothers was conducted. They were asked to complete the questionnaire and provide feedback on the structure and nature of questions. This pilot study is essential for assessing the validity of the instrument and evaluating the level of difficulty of the questions posted. The estimated time spent by each respondent for the whole questionnaire was recorded. In addition, the other aspects that might interfere with the process of data collection and analysis was also identified. This pilot study is also designed to examine the reliability test (internal consistency) using Cronbach’s alpha. Cronbach’s α was used to assess the homogeneity of the questions for internal consistency within the test. Researchers have checked Corrected Item Total Correlation (CITC) and made a comparison of the Cronbach alpha if any item was deleted. The overall value of Cronbach Alpha assessment instruments for the pilot study exceeded the reference value (α = 0.8) [20]. The final version of the questionnaire is provided in online appendix 1.

### Data collection

The study was ethically approved by the Medical Research and Ethics Committee, Ministry of Health Malaysia with code NMRR-20-2503-57,029 (IIR). An informed consent was acquired before each respondent administered the questionnaire online. Before entering the study, all potential respondents were introduced with the study purpose and contents. After a comprehensive explanation of the study and acknowledgement of the anonymity and de-linkage principle together with the other ethical considerations by the respondents, they carried on fulfilling the questions online based on their own willingness. This was implied consent - respondents who click on “I agree” and complete the survey deemed to have consented to participating. This questionnaire was available in Malay language because most of the respondents were Malays, and they understood and preferred the language.

### Data analysis

The results for each questionnaire were compiled in Survey Monkey, an online survey program, and the total responses to each question were compiled for analysis. The data from this questionnaire was analysed using the Statistical Package for Social Sciences (SPSS) version 22. Categorical variables were summarized as numbers and percentages, whereas normally distributed continuous variables were presented as means and standard deviations. *p-value* less than 0.05 was considered statistical significant.

## Results

### Respondents’ characteristics

Table 1 shows that all respondents were wet nurses who lived in Selangor. Variables of the study were age, marital status, several children are breastfed, residence, education level, occupation, and total income. The respondents in this study were 21 to 50 years old. The results showed that all respondents were married (100.0%). In terms of education, the respondents were examined from various educational backgrounds: *Sijil Pelajaran Malaysia* (SPM), Certificate, Diploma, Bachelor’s Degree, Master’s Degree, and the highest level of Doctor of Philosophy. The study showed that respondents with a bachelor’s degree outperformed respondents from other education levels of 48.0%. In terms of employment, 37.0% of the respondents were civil servants. While 32.0% of respondents worked in the private sector, and only 3.0% of respondents were students. Therefore, most of the respondents in this study consist of professionals who have monthly income that ranged from RM3001 to RM6000 (40.0%). Whereas 31.0% of respondents had RM1001 to RM3000 salary, and only 17.0% of respondents had below RM1000 salary.

**Table 1:** Socio-demography of respondents

Item	Respondent	
	Frequency (n)	Percentage (%)
**Age (year)**		
21–30	30	30.0
31–40	64	64.0
41–50	6	6.0
**Marriage status**		
Single	0	0
Married	100	100.0
Divorce	0	0
**Residence (district)**		
Kuala Selangor	3	3.0
Kuala Langat	5	5.0
Sepang	8	8.0
Hulu Langat	26	26.0
Gombak	12	12.0
Petaling	28	28.0
Klang	18	18.0
Sabak Bernam	0	0
Hulu Selangor	0	0
**Education leve**		
SPM	7	7.0
Certificate	2	2.0
Diploma	20	20.0
Bachelor	48	48.0
Master	19	19.0
PhD	4	4.0
**Occupation**		
Civil sector	37	37.0
Private sector	32	32.0
Self-employed	13	13.0
Housewife	15	15.0
Student	3	3.0
**Income**		
Below RM1000	17	17.0
RM1000-RM3000	31	31.0
RM3001-RM6000	40	40.0
RM6001-RM9000	10	10.0
RM9001- above	2	2.0

### Wet nursing practices

#### Influence factor to a wet nurse

Table 2 shows the frequency, percentage, and mean of each item or questionanswered by the respondents. Analysis made based on the mean showed that the item with the highest mean value was the third item, “I breastfeed other milk children because I want to help mothers who do not have enough milk” by reaching mean = 3.36. Followed by the first item, “I breastfeed milk children because I have more milk” (mean = 3.18) and the fourth item, “I breastfeed milk children because their mothers are suffering from health problems preventing her from breastfeeding” (mean = 3.01). While the least favourite item was the second item, “I breastfeed other milk children as a source of income” (mean = 1.41). On average overall, although the number of respondents who agreed is higher than disagreed, it is observed that some respondents decided to make breastfeeding someone else’s children as a source of income.

**Table 2:** Influence factors to wet nurse

No	Influence factors in breastfeeding others child	Strongly disagree	Disagree	Agree	Strongly agree	Mean ± SD
		n	n	n	n	
		%	%	%	%	
1	I breastfeed another child because I have more milk	4 4.0	12 12.0	46 46.0	38 38.0	3.18 ± 0.80
2	I breastfeed another child as a source of income	60 60.0	39 39.0	1 1.0	0 0.0	1.41 ± 0.51
3	I breastfeed another child because I want to help mothers who do not have enough milk	2 2.0	6 6.0	46 46.0	46 46.0	3.36 ± 0.69
4	I breastfeed another child because her/his mother is suffering from health problems preventing her from breastfeed (maternal debility)	5 5.0	22 22.0	40 40.0	33 33.0	3.01 ± 0.87

Note: ‘ƒ’: frequency of respondents; ‘%’: Percentage of respondents

#### Frequency of wet nursing

This study shows that a total of 42 respondents had breastfed at least one someone else’s child. While one of the respondents stated that she had nursed a total of 10 someone else’s children. Table 3 also shows a total of 237 children have been breastfed by the respondents (*N* = 100). Of these, only 78.5% were breastfed five times, while 21.5% were breastfed less than five times. The findings show that there is still a wet nurse who did not achieve the conditions that can be categorized as a milk child that “the number of milk shall be five feedings” as determined by the National Muzakarah of Malaysia [21].

**Table 3:** Frequency of wet nursing

No.	Total of children	Total of respondents	Frequency of wet nursing				Total of children
			Less than five times		Five times or more		
			n	%	n	%	
1	1	42	10	23.8	32	76.2	42
2	2	17	7	20.6	27	79.4	34
3	3	22	19	30.6	43	69.4	62
4	4	10	9	22.5	31	77.5	40
5	5	2	3	30.0	7	70.0	10
6	6	4	0	0.0	24	100.0	24
7	7	1	0	0.0	7	100.0	7
8	8	1	2	25.0	6	75.0	8
9	10	1	1	10.0	9	90.0	10
	**TOTAL**	**100**	**51**	**21.5**	**186**	**78.5**	**237**

#### Method of feeding

Table 4 shows the feeding method that has been used to feed someone else’s child. Most respondents feed children using bottles (82.0%), followed by direct breastfeeding (58.0%). On the other hand, 11.0% of respondents used cups and 10.0% used syringes. Only 8.0% of respondents used tube (Supplemental Nursing System) to help them deliver milk to the children. This indicates that almost all respondents understand that the practice is permissible by legislation, and that there is the milk extraction condition, in which the sipped milk must reach the baby’s stomach.

**Table 4:** Method of feeding

Method of feeding	Frequency (n)	Percentage (%)
Direct breastfeeding	58	58.0
Using a cup	11	11.0
Using a bottle	82	82.0
Using a syringe	10	10.0
Using a Supplemental Nursing System (SNS)	8	8.0
Others method	7	7.0

#### Providing breast milk with the combination of many other foods and beverages

Table 5 shows most of the respondents provided breast milk without any other additional food. However, this study also showed that 17.0% of the respondents mixed their breast milk with a variety of different foods, respectively, from the highest percentage to the lowest, includes porridge (17.0%), formula milk (14.0%), biscuits (14.0%), cereal (7.0%), fruit/vegetable puree (3.0%) and breast milk from another mother (1.0%).

**Table 5:** Breast milk combined with other foods and beverages

Food and other beverages	Frequency (n)	Percentage (%)
Breast milk only	57	57.0
Breast milk + another mother’s breast milk	1	1.0
Breast milk + formula milk	14	14.0
Breast milk + cereal	7	7.0
Breast milk + porridge	17	17.0
Breast milk + biscuit	14	14.0
Breast milk + fruit/ vegetable puree	3	3.0

### Issues related to wet nursing

#### Payment received during wet nursing

Based on Table 6, the majority of respondents did not receive any payment or charged any fees for wet nursing (94.0%). However, some respondents received a payment of RM100 (4.0%), RM1 (1.0%) and RM50 (1.0%) for wet nursing. This clearly shows that almost all respondents voluntarily breastfed other children at no charge.

**Table 6:** Total payment received during wet nursing

No	Total payment (RM)	Frequency (n)	Percentage (%)
1	No charge	94	94.0
2	1	1	1.0
3	50	1	1.0
4	100	4	4.0

#### Affinity relationships between mother and milk child

Table 7 shows that the majority of respondents (93.0%) recognized their milk children. However, it also showed that the remaining 7.0% could not identify their milk children, which is somehow worrying. Meanwhile, 13.0% of respondents did not know the identity of their milk children. When asked about their relationships with the milk children, the majority of them were still in contact (95.0%) and knew where they live (91.0%). Some who had lost contact with their milk children did not have any information regarding the presence of their infants/milk children.

**Table 7:** Affinity relationships between wet nurse and milk child

No.	Affinity relationships	Yes		No	
		n	%	n	%
1	Do you recognize your milk child?	93	93.0	7	7.0
2	Do you and your family know the identity of the milk child?	87	87.0	13	13.0
3	Are you still in touch with your milk child?	95	95.0	5	5.0
4	Do you know where / residence milk children?	91	91.0	9	9.0

Note: ‘ƒ’: frequency of respondents; ‘%’: Percentage of respondents

#### The practice of wet nursing documentation

Questionnaires were distributed to obtain information on the importance of keeping wet nursing data records. Table 8 shows that not all respondents recorded the information of their milk children. Only 72.0% of them documented data on their milk children’s background. When they were asked about the importance of recording data on their milk children, there are still 15.0% of respondents who thought that the data were unnecessary to record. Although the majority of respondents felt that it should be recorded (85.0%).

**Table 8:** The importance of recording breastfeeding information

No.	The importance of wet nursing documentation	Yes		No	
		n	%	n	%
1	Did you record your infant’s milk child background data?	72	72.0	28	28.0
2	Do you feel the need to record your infant’s milk child background data?	85	85.0	15	15.0

Note: ‘ƒ’: frequency of respondents; ‘%’: Percentage of respondents

Nevertheless, the awareness of keeping information on milk children should be raised among the breastfeeding mothers to prevent any undesireable situations following wet nursing activity; the woman and milk child might be settling elsewhere and no longer meet. They may even be living outside the state or perhaps residing abroad and so on. This situation can result in disconnected and inaccessible contact.

## Discussion

Here we commence our discussion of the results that the practice of wet nursing in Selangor is on the rise. This practice is due to the emergence of awareness among the public about the importance and advantages of breastfeeding. This was concluded based on the profound findings of the percentage and frequency of wet nursing practice by Muslim women in Selangor. In the present study, mothers aged < 41 years old (94.0%) had a higher ratio of having a wet-nurse than older mothers (6.0%). This indirectly implies an increase of wet nursing practice over the years. However, in Turkey, the practice of wet nursing has decreased but not thoroughly forgotten where some people still practice it [22]. The finding that most of the wet-nurses (66.0%) were among their own relatives shows that wet nursing is still practiced among relatives. Some reasons why wet nurses were chosen from relatives or familiar people were to ensure cleanliness and proper nutritions; wet-nurse’s greater love, compassion and mercy; and the ease to obtain breast milk [23–25].

Based on research findings, we concluded that respondents had extensive experience related to wet nursing. This indicates that respondents have massive experience in marriage and the intricacies of breastfeeding. Many respondents had experienced breastfeeding almost ten children and most of these children are breastfed five times or more. When the non-biological children are breastfed for five times or more within the first 2 years, they indirectly established a milk kinship between them. Therefore, the mother becomes *mahram* or *haram* (illegal) to be married to the child she nursed [12]. The term *mahram* is used to denote a level of relationship between close family members, i.e., those with whom the hijab does not have to be observed [26].

However, there were still some respondents who breastfeed their milk children less than five times in which based on the legislation, it does not qualified to be classified as a suckling. This indicates that there were still some respondents who did not understand that by law, breastfeeding will only be called breastfeeding if it is done for at least five times. According to Zulkifli [27], it would condemn the child-mother relationship when the total breastfeeding is only five times full. However, this is contrary to the National Fatwa Councils of Malaysia’s [21] opinion on the conditions of fullness that were never discussed but were only expressed as fivefold. Siti Zainab [28] stated that there was no language or legal standard for it. Therefore, one possible method is to adhere to the custom or recognition of the mother [29–32]. Besides, the baby’s need for breast milk also varies depending on the age, stomach capacity, level of activity, and sleeping patterns. According to Nazirah and Zaharah [33], infants are given the ability to determine the level of their food. Naturally, babies will show signs when they are hungry and stop by themselves when they are full.

In the case of feeding method, the majority of respondents knew that there were two methods of feeding agreed by the majority of Scholars to make the baby *mahram* through breast milk. They were feeding directly from the breast and breastfeeding indirectly with the aid of breastfeeding such use tubes, bottles, Supplemental Nursing System (SNS) and the like with the proviso that the baby milk must reach the stomach [21]. Babies who are not fed at the breast can be fed by nasogastric or orogastric tube, syringe, or dropper (not more than 0.5 ml at a time), spoon, cup or direct expression into the baby’s mouth. The need for alternative feeding methods and the most suitable method should be individually assessed for each woman and baby [34]. No matter which method used to feed breast milk to babies, the argument is that breast milk is a staple food for babies to grow flesh and for bone lengthening, hence, it is just as similar if babies feed directly from the breast. However, if the procedure is done by injecting milk through the anus, liquid milk dripping into the eyes, ears, or in the wounds of the body, then the baby is not valid as a suckling [21]. Fatwa Selangor [35] explained that breast milk mixed with other ingredients is the same as breast milk without them because it already meets the conditions prescribed by the Islamic Law for a milk child. The results showed that many respondents mixed their breast milk with a variety of other foods such as formula milk porridge, biscuits, and cereal. Breast milk was also mixed with pureed fruits/vegetables. However, most respondents provided only breast milk without mixing it with other foods.

In Malaysia, the demand and supply of breast milk are becoming increasingly widespread and widely accepted [36]. In fact, some women who have a surplus of milk were prepared to sell it to those in need. From this research, most respondents voluntarily provided their milk without any charges. Nonetheless, a small percentage of respondents make wet nursing practice as a source of income. This proves that the media [37] reported correctly, where some women offer their breast milk for money. This development can lead to an unhealthy phenomenon as it could be considered a lucrative career. We hope that the authorities will look into this activity seriously and take more aggressive steps to curb it. The 153rd Fatwa Census issued by the Federal Territory Mufti Office also made it clear that the legal sale of breast milk is not allowed. This is because the selling of breast milk could result in the mixing of *mahram* and bringing Muslims into doubt and illicit affairs.

It is crucial to document wet nursing practices to avoid any misunderstanding between a breastfeeding mother and her milk child and their descendants. Although most respondents took their initiative to document their wet nursing data, there were still some respondents who felt that documenting was unnecessary. A study that was conducted by Siti Fatimah et al. [38] shows that documenting evidence of breastfeeding by individual bodies such as the National Registration Department is an essential step in harmonizing the Islamic family system. This was also touched upon by the Federal Territory Mufti, Dr. Zulkifli al-Bakri [27], who recommended the creation of a special card for each wet-nursed infant to avoid problems like milk kinship relationship misunderstanding. In the absence of any specific record, many issues might raise in terms of Islamic law as well as health. According to Azizah [39] an adoptive mother should provide a witness or a proof that says that the she has breastfed the child to avoid any problems later. It thus leads to the issue of notes or records as a wet nurse as proof of the practice. In addition, each milk donor should write on the bottle the name of the contributing mother. A total of five times as much as possible will convince the child and the mother of their relationship based on the majority of Scholars. Most women in Turkey prefer to become wet nurses or do milk sharing if appropriate, but were reluctant to donate their milk to human milk banks, mainly because of religious concerns [22].

Breastfeeding plays a vital role in the development of love, attachment and dependence on the mother, and has been proved by Muslim scholars [40–43] and non-Muslim researchers [44–50]. This is consistent with the findings of this study in which almost all the items involving the practice of wet nursing to convict lineage, noting the high percentage and mean. Most respondents were aware of the practice. It has also been supported by the findings by Zanariah et al. [50], which states that the awareness of the Muslim community in Selangor on the effects and laws of breastfeeding, especially on the aspects of genitalia, lineage, and marriage is at an outstanding level.

### Strengths and limitations of the study

The strengths of this study are that it provides novel information regarding wet nursing trends, influences, and practices among a sample of Muslim mothers living in Selangor. This study also used primary data that should consider the strength of this study. However, there can be a recall bias among the respondents since the information was collected retrospectively, one to 2 years after the wet nursing practice completed. Due to that, we cannot rule out the potential recall biases in the responses given in the interviews. In this sense, it is possible that the reported breastfeeding rates overestimated the actual rates.

## Conclusion

In conclusion, this novel study shows that there are a lot of useful information that indicates the wet nursing practice among Muslim mothers in Selangor. However, the issue of breastfeeding has uncovered several findings, including the role of government in facilitating breastfeeding documentation to the public and researchers being able to identify informal documented situations by those who have adopted breastfeeding practices. In general, respondents are aware of the fact that it is an important personal documentation to facilitate any future issues. The public also supports the fact that there is a formal documentation by the local authorities to ensure the rights and well-being of the Islamic community in the future. It indirectly implies the need for such practices to be recorded. It will help to safeguard the welfare of the breastfed children, breastfeeding, mother’s milk, and of course, biological family of the milk child. The documentation is suggested to prevent any difficulty in the future to prove mahram relationship through breast milk. Researchers have also suggested some improvements on the current practices through legislation, policies, or mechanisms in the implementation documentation wet nursing practices that apply to the Muslim community in the state. However, it is expected that similar efforts are also conducted in other states in Malaysia as an initiative towards keeping the lineage and descendant generations to come because this situation does not happen only in Selangor but also includes other states as well.

## Supplementary Information

**Additional file 1.**

## Data Availability

The datasets generated and/or analysed during the current study are not publicly available due to the sensitive nature of the information of the respondents provided but are available from the corresponding author on reasonable request. This is to protect and maintain respondents’ anonymity and confidentiality. The data are kept saved in order not to expose the feelings of the respondents to the public.

## Abbreviations

CITC: Corrected item total correlation
SPSS: Statistical package for social sciences
SPM: *Sijil Pelajaran Malaysia*
PhD: Doctor of philosophy
SNS: Supplemental nursing system
