# Supplementary material for: A cross-sectional study on the practice of wet nursing among Muslim mothers
Source: BMC Pregnancy Childbirth. 2021 Jan 21;21:68. doi: 10.1186/s12884-021-03551-9 (PMC7819175; doi:10.1186/s12884-021-03551-9)
Supplement: Supplementary file 1 — Additional file 1. [file 12884_2021_3551_MOESM1_ESM.pdf]

ID Number:______

QUESTIONNAIRE

PRACTICE OF WET NURSING AMONG MUSLIM MOTHERS

To the respected mothers, the Selangor Islamic Religious Council (MAIS) has given responsibility to us (investigators) to conduct investigations to learn more about the practice of the child's breastfeeding among the Islamic community in the state of Selangor. Thus, we strongly need the cooperation of the mothers to gather for the purpose of collecting data to improve this investigation. All information is confidential and it is only used for research purposes only. Please cooperate with the mothers to respond to these surveys and be able to return this form with immediate rate to any member of this group of researchers.

THIS QUESTIONNAIRE CONSISTS OF 2 PARTS:

- **Part A**: Personal information
- **Part B**: Wet nursing practice

The cooperation given is very appreciated and expressed thanks.

Investigators:

1. Associate Prof Dr Nurhidayah Binti Muhammad Hashim (UiTM) (Principle Investigator)
2. Associate Prof Dr Salasiah Hanin Binti Hamjah (UKM)
3. Associate Prof Dr Latifah Binti Abdul Majid (UKM)
4. Associate Prof Dr Zuliza Binti Kusrin (UKM)
5. Associate Prof Dr Rafeah Binti Saidon (UiTM)
6. Dr. Nora'inan Binti Bahari (KUIS)
7. Mrs. Norsyamlina Binti Che Abdul Rahim (MOH)
8. Mr. Muhamad Zariff Bin Ilias (UiTM)

PART A: PERSONAL INFORMATION

Instructions: Please tick (√) on the answers

1. **Age**
( ) 20 years and under
( ) 21-30 Year
( ) 31-40 Year
( ) 41-50 Year
( ) 50 years and older

2. **Marital Status**
( ) Bujang
( ) Married
( ) Janda/Balu

3. **Number of wet nursing children**

Please specify:
____

4. **Place of residence/area**
( ) Sabak Bernam
( ) Hulu Selangor
( ) Kuala Selangor
( ) Kuala Langat
( ) Sepang
( ) Hulu Langat
( ) Gombak
( ) Petaling
( ) Sound
( ) Other:_________

5. **Education Level**
( ) SPM
( ) Diploma
( ) Bachelor Degree
( ) Bachelor Degree
( ) Doctoral degree
( ) Other: ____________

6. **Occupation**
( ) Civil sector
( ) Private sector
( ) Self-employed
( ) Housewife
( ) Student

7. **Income**
( ) Below RM1000
( ) RM1000-RM3000
( ) RM3001-RM6000
( ) RM6001-RM9000
( ) RM9001- above

PART B: WET NURSING PRACTICE

Please circle the appropriate answer based on the scale below.

Strongly Disagree	Disagree	Agree	Strongly Agree
1	2	3	4

B: WET NURSING PRACTICE

Please tick (√) on the answers

B1. Influence factors to wet nurse (tick only one)

1	I breastfeed another child because I have more milk	1	2	3	4
2	I breastfeed another child as a source of income	1	2	3	4
3	I breastfeed another child because I want to help mothers who do not have enough milk	1	2	3	4
4	I breastfeed another child because her/his mother suffering from health problems preventing her from breastfeeding (maternal debility)	1	2	3	4

B2. The frequency of wet nursing (tick only one)

**Car**	**Child Susuan**	**Less 5 times**	**5 times and more**
1	First child		
2	Second child		
3	Third child		
4	Fourth child		
5	Fifth child		
6	Sixth child		
7	If more please specify		

B3. Methods of feeding (can tick more than one)

( ) Directly from breasts (direct breastfeeding)
( ) Using a cup
( ) Using a bottle
( ) Using a syringe
( ) Using a Supplemental Nursing System (breastfeeding aid)
( ) Other: (please specify)_______________________________

B4. Have you ever been combined breast milk with other foods? If yes, please tick (√) the following statements (may indicate more than one)

( ) Breastmilk only
( ) Breastmilk + another mother’s breastmilk

( ) Breastmilk + formula milk
( ) Breastmilk + cereal
( ) Breastmilk + porridge
( ) Breastmilk + biscuit
( ) Breastmilk + fruit/ vegetable puree

B5. OTHERS RELATED WET NURSING PRACTICE

Total payment was received during wet nursing
( ) No charge
( ) RM1
( ) RM50
( ) RM100

Do you recognize your milk child?
( ) Yes ( ) No

Do you and your family know the identity of the milk child?
( ) Yes ( ) No

Are you still in touch with your milk child??
( ) Yes ( ) No

Do you know where/residence milk children?
( ) Yes ( ) No

Did you record your infant's milk child background data?
( ) Yes ( ) No

Do you feel the need to record your infant's milk child background data?
( ) Yes ( ) No

- Thank you for the cooperation -
